# Differential Angular Expansion in Perceived Direction in Azimuth and Elevation Are Yoked to the Presence of a Perceived Ground Plane

**DOI:** 10.3390/vision2020017

**Published:** 2018-03-24

**Authors:** Frank H. Durgin, Umi I. Keezing

**Affiliations:** Department of Psychology, Swarthmore College, Swarthmore, PA 19081, USA

**Keywords:** visual perception, space perception, angular expansion

## Abstract

It has been proposed that perceived angular direction relative to straight-ahead is exaggerated in perception, and that this exaggeration is greater in elevation (or declination) than in azimuth. Prior research has suggested that exaggerations in elevation may be tied to the presence of a visual ground plane, but there have been mixed results across studies using different methods of dissociation. In the present study, virtual environments were used to dissociate visual from gravitational upright while human participants (N = 128) made explicit angular direction judgments relative to straight ahead. Across these experimental manipulations, observers were positioned either upright (Experiments 1A and 1B) or sideways (Experiment 2), so as to additionally dissociate bodily orientation from gravitational orientation. In conditions in which a virtual environment was perceived as containing a level ground plane, large-scale exaggerations consistent with the visually-specified orientation of the ground plane were observed. In the absence of the perception of a level ground plane, angular exaggerations were relatively small. The ground plane serves as an important reference frame for angular expansion in the perceived visual direction.

## 1. Introduction

The observation that visual direction (relative to straight-ahead) is exaggerated has been independently documented at least three times in real outdoor environments. Higashiyama initially described this as an exaggeration of perceived visual angle [1]. He observed that the perceived visual angle for large-scale vertical extents was greater than that for horizontal extents in a manner that might account for the large-scale horizontal-vertical illusion (HVI) [2,3,4]; Li and Durgin discovered that exaggeration in downhill slant perception was amplified by conditions that yoked it to an exaggerated perception of downward gaze direction or angular declination [5]. Durgin and Li ultimately showed that these exaggerations in perceived angular declination could quantitatively account for long-standing observations of explicit ground distance underestimation [6,7,8]. Indeed, Foley and colleagues [9] had interpreted underestimates of ground distance using the assumption that the effective (but not perceived) angular size of the ground extents, from the point of observation, was differentially exaggerated in azimuth and in elevation.

Non-verbal distance matching tasks, verbal distance estimation tasks, verbal angular estimation, and non-verbal, but explicit horizontal/vertical direction bisection tasks all converge on an estimate of a 1.5 gain in perceived visual direction in elevation relative to straight-ahead [1,6,8,9]. Despite being discovered in outdoor environments that involved ground planes, the possible role of the ground plane in producing this very large exaggeration in perceived elevation has not been entirely clear, though it is implicated in at least one study [4].

A series of follow-up studies conducted in our laboratory with outdoor environments indicated that the exaggeration in perceived direction in azimuth had a gain of about 1.25, and was clearly less than the 1.5 gain observed for elevation [8,10]. Further studies suggested that the gain differential between azimuth and elevation might be tied predominantly to the orientation of the visual ground plane rather than the orientation of the observer [4]. There is some prior evidence that, for an observer lying on his or her side, the anisotropy between elevation and azimuth (as measured by distance-matching tasks) disappears [11]. However, it remains possible that details of how re-orientation was accomplished mattered. Elevating sideways observers to normal eye-height [4], may provide more access to ground-based information than is available when sideways observers are at a much lower viewing position [11].

The primary purpose of the present study was to examine the possible role of the perceived ground plane in establishing the presence of angular expansion in judgments of angular direction. Because most prior studies depended primarily on uncontrolled (i.e., real-world) environments, we sought to measure the influence of the ground plane on perceived angular direction using virtual environments. Although some previous studies have used virtual environments in addition to real environments to provide a re-oriented ground plane [4], they used exclusively implicit measures, and have not compared ground-plane conditions to conditions in which the ground plane was simply absent.

This is important because some indoor studies of perceived visual direction that do not include a ground plane have seemed to suggest that the exaggeration of azimuthal direction is somewhat less than that implied by outdoor studies [12,13,14], though others have proposed alternative reinterpretations of this data [8]. The methods used in these various indoor studies differed substantially from each other and from the outdoor studies conducted on ground surfaces. It is thus possible that some of the variance in estimates between these studies is due to changes in the measures used as well as or instead of the differences in the environments used.

In the present study, we sought to have participants directly estimate the perceived direction to targets in virtual environments such that we could better control both the orientation of the ground plane and also its presence or absence under otherwise similar viewing conditions. Previous studies that have used explicit numeric estimates of visual direction or visual angle [1,5,6,8], have shown remarkable consistency between explicit verbal estimation and implicit measures, such as of perceived relative size or distance [4,9]. Thus, numeric estimates represent an important converging measure that can be added to evidence from existing studies reporting the effects of virtual world rotation on the HVI [4] that can be interpreted in this framework.

Note that across all of the experiments reported here, all presented angles could be described as being in an azimuthal direction relative to the gravitational upright. In Experiment 1A, the observer and world reference frames corresponded with gravity. In Experiment 1B and 2, the visual world reference frame is sometimes reoriented, and in Experiment 2, the observer is reoriented.

## 2. Experiment 1

Experiment 1 consisted of two different kinds of manipulation. All participants were seated upright, and all angle estimates were made with respect to a (gravitationally) vertical edge that was straight ahead. All participants made one set of angular estimates of a black ball at a constant distance and a variable azimuthal position with respect to an upright environment consisting of a ground plane and a distant pole representing the straight ahead.

**Figure 1:**
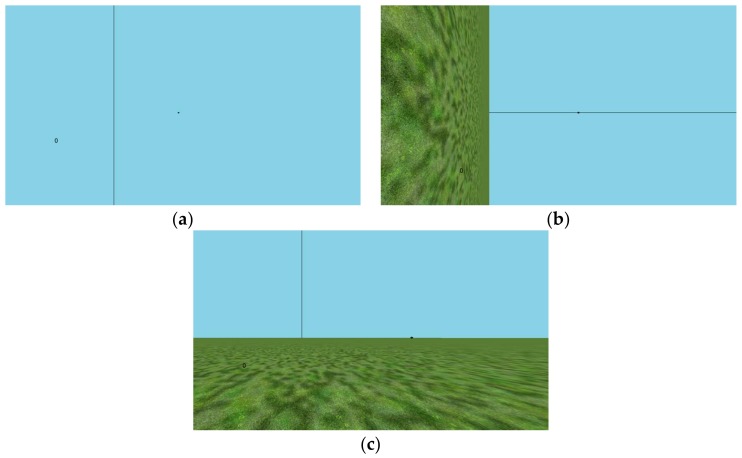
(**a**) The experimental condition in Experiment 1A, in which a vertical edge is straight ahead and there is no ground plane. The target is to the right. Participants sat to the left of the center of the screen to allow a greater field of view to the right, where stimuli were presented. A small zero is present to show where the spoken response appeared once typed in by the experimenter. (**b**) shows the experimental condition in Experiment 1B, in which the ground plane orientation is reoriented by 90° about straight-ahead. (**c**) shows the baseline comparison condition, for both Experiments 1A and 1B, in which estimates of azimuth were collected with a (gravitationally) horizontal ground plane present. We refer to this as the upright or normal ground plane.

Participants in Experiment 1A also made estimates of azimuthal angle with the ground plane removed entirely, so that only the distant pole was visible as shown in Figure 1 in panel a. In Experiment 1B, in addition to a normal scene with a ground plane, all participants made estimates of the same actual ball positions, but with a rotated virtual world in the background, so that the actually-azimuthal position appeared to vary in elevation relative to the simulated ground plane as shown in Figure 1 in panel b. The upright condition is shown in Figure 1 in panel c.

We expected that removing the ground plane entirely would reduce the amount of angular exaggeration, but that rotating the virtual environment would increase the amount of angular exaggeration by rendering azimuth (with respect to the observer and to the gravitational upright) as elevation (with respect to the simulated environment).

### 2.1. Methods

All procedures reported here were approved by a local Institutional Review Board at Swarthmore College. All participants gave informed consent prior to participation.

#### 2.1.1. Participants

There were a total of 64 Swarthmore College undergraduate students who successfully completed the experiment. Half were in Experiment 1A and half in Experiment 1B. Three additional participants were replaced because of technical problems or failure to follow instructions. Participants were required to have normal or corrected-to-normal vision in the absence of glasses.

#### 2.1.2. Design

Half of the participants in each experiment completed their estimates in normal upright world first and then completed the other condition (either the world without a ground plane (Expt. 1A) or the sideways world (Expt. 1B). The remaining participants were assigned to the opposite order. Seventeen different angles, ranging from 3° to 51° by 3° increments, were tested in random order in each condition. After an initial practice block in the first condition to be tested, two blocks of 17 experimental trials were collected in the first condition followed by two blocks of 17 trials in the second condition tested. For purposes of analysis, a single parameter (gain) was computed for the data for each participant in each condition, assuming an intercept of 0°.

#### 2.1.3. Apparatus and Stimuli

A polarization-retaining back-projection screen was used to present a stereoscopic virtual scene. The projector was a ProPIXX with a resolution of 1920 × 1080 pixels projected to a size of 2.56 × 1.44 m. An active circular polarizing filter (DepthQ, Lightspeed Design Inc., Bellevue, WA, USA) allowed the 120 Hz projector signal to be split into 60 Hz left- and right-eye streams of images. Vizard 5.0 software (WorldViz, Santa Barbara, CA, USA) was used to generate the virtual environment. The viewer wore passive polarizing glasses and was held stationary in a chin and forehead rest so that viewing position was fixed at a viewing distance of 0.64 m from the screen, and 0.5 m to the left of center of the screen. The stereo simulation included a parameter for interpupillary distance, measured with a Shin-Nippon PD-82, so that the stereoscopic geometry of the scene would be accurate for each observer. The available field of view to the right of straight-ahead was 70°.

The virtual environments, as depicted in Figure 1, each included a distant pole (2 m diameter; 500 m distant) that served as the reference for straight ahead, and a black ball (0.1 m diameter; 8 m distant) that was suspended at eye level.

#### 2.1.4. Procedure

The angular direction estimation task was explained as referring to the apparent direction to the black ball relative to straight ahead. The experimenter reminded participants that angular direction could range from 0 (for straight ahead) to 180° (directly behind them). They were encouraged to report the perceived direction and to be as precise as possible in their estimates. There was no other training or feedback. On each of the 85 trials (17 practice), participants reported perceived direction orally in degrees. Their estimate was typed in by an experimenter and presented as text superimposed on the scene, so that participants could confirm that an accurate recording of their estimates was made. The screen was blanked for about 1.5 s between trials after the response was accepted, before the next trial. The ground texture was randomly shifted while the screen was blanked between each trial so that ground texture features could not serve as a reference.

#### 2.1.5. Analysis

Our primary dependent measure was a single parameter representing the gain of angular expansion in each condition tested. This was computed for each participant using the slope of the best fit line for their estimates that went through the origin (i.e., the intercept was forced to be zero). We had reason to expect that the frame of the screen might produce non-linear response effects [14], but these were not of interest in the present investigations; this is why statistical analyses were limited to the underlying angular gain relative to the origin. Response compression (central tendency) can produce a positive intercept and reduced effective slopes in participant responses [10]. Given that a zero azimuth would necessarily produce a response of zero, the theoretical parameter under investigation (angular gain rather than estimate gain) is probably best estimated by assuming a zero intercept [8]—though 2-parameter models have often been used in the past [5,6]. The mean coefficient of determination (*R^2^*) for the individual fits in Experiment 1 was 0.98. A Shapiro-Wilk normality test conducted on the resulting gain values for Experiments 1A and 1B was consistent with a normal distribution, *W* = 0.99, *p* = 0.711.

### 2.2. Results and Discussion

#### 2.2.1. Removing the Ground Plane

In Experiment 1A, an ANOVA with environment (ground plane present or absent) as a within-subject variable, and order (present first or absent first) as a between-subject variable showed a main effect of ground plane as expected, *F*(1, 30) = 10.4, *p* = 0.002, *η^2^* = 0.06. In the absence of a ground plane, the mean angular gain in azimuthal direction was only 1.15 (95% CI [1.04, 1.24]), whereas it was higher when a ground plane was present, 1.26 (95% CI [1.18, 1.34]). These observations are consistent with the importance of the ground plane.

**Figure 2:**
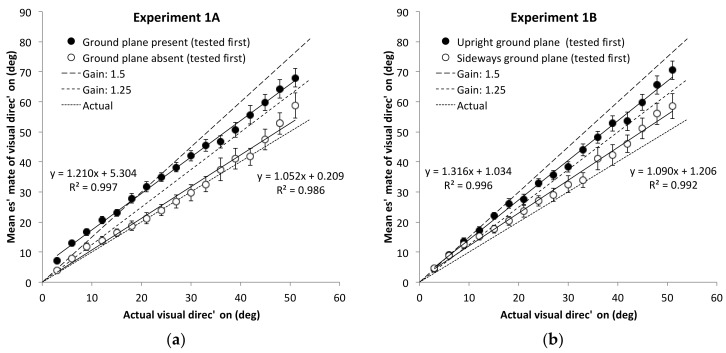
Mean direction estimates (with standard error bars) for the upright (normal) ground plane condition (black circles) and for the unique experimental conditions (white circles) for (**a**) Experiment 1A (no ground plane) and for (**b**) Experiment 1B (sideways ground plane). Only data from the first-tested conditions for each participant are shown in each plot. Note that the best-fit lines shown here are 2-parameter fits, including an intercept, whereas the gain values used for our statistical analyses were based on a single parameter fit (slope only).

There was an order effect as well, however, *F*(1, 30) = 6.25, *p* = 0.018, *η^2^* = 0.14, and this order effect was suggestive of contamination of scaling between the two conditions based on the order in which they are done (i.e., the gain observed in the second condition tended to be biased toward the gain of the first). It therefore seemed prudent to re-examine the first condition tested as a between-subject design. A between-subject ANOVA with environment as the only factor, that was limited to the first condition tested for each participant indicated that the gain for the ground-plane present condition, *M* = 1.36, 95% CI [1.24, 1.49], was reliably higher than the gain in the ground-plane absent condition, *M* = 1.05, 95% CI [0.89, 1.21], *F*(1, 30) = 10.4, *p* = 0.003, *η^2^* = 0.25. The mean angular estimates are shown in the left panel of Figure 2. The magnitude of angular expansion in the ground-plane present condition is consistent with the amount observed with explicit estimates of azimuth direction in outdoor environments. The significant reduction in the gain when the ground plane was removed is consistent with the interpretation that the ground plane is extremely important in determining the magnitude of the gain factor in perceived azimuth.

Although the mean gain estimate with the ground plane present is nominally higher than 1.25, and there is some suggestion of curvature in the estimate function, a 2-parameter least-squares fit to the mean estimates had a gain of 1.21 and an *R*^2^ of 0.997. The intercept of this fit was 5.3°. As noted above, a positive intercept may result from response compression, and is not of current theoretical interest.

#### 2.2.2. Reorienting the Ground Plane

In Experiment 1B, an ANOVA with environment (ground plane upright (normal) or sideways) as a within-subject variable, and order (upright first or sideways first) as a between-subject variable indicated a reliable interaction between order and environment, *F*(1, 30) = 6.36, *p* = 0.017, *η^2^* = 0.02. Although the original expectation had been that turning the ground plane sideways would increase the gain of estimates toward 1.5 (consistent with judgments of elevation), this did not occur. When the normal ground plane was tested first, the angular gains found in the normal condition, *M* = 1.35, 95% CI [1.21, 1.48], were essentially identical to those observed in Experiment 1A, and were reliably greater than those found thereafter for the sideways condition, *M* = 1.16, 95% CI [1.05, 1.27], *t*(15) = 4.50, *p* < 0.001, *d* = 1.12.

Although this result appears to be inconsistent with our experimental hypothesis that turning the world sideways would increase the gain (i.e., make it approximately 1.5, consistent with angular expansion of elevation in the sideways environment), here the use of stationary participants may have been consequential. Our own conscious perceptual impression of the sideways world was not consistent with what the underlying geometry specified. Specifically, the “wall” formed by the ostensible ground plane did not appear to be parallel to the line of gaze, nor did it even appear to be planar. Consistent with other reports of depth compression in virtual environments e.g., [15], the vertical ground plane instead appeared convexly curvilinear in depth. This led us to hypothesize that the upright ground plane appeared flat and parallel with gaze because of expectancies about ground surfaces. Experiment 2 followed up on these observations.

### 2.3. Interim Conclusions

In Experiment 1A we saw evidence that the magnitude of angular expansion in azimuth depended on the presence of a ground plane: Angular expansion in azimuthal direction was much reduced in the absence of a ground plane. In Experiment 1B, when the simulated sideways ground plane failed to have the appearance of a ground plane, we again saw a reduction in angular expansion relative to the normal ground plane condition.

## 3. Experiment 2

Reasoning that a sideways ground plane might be more recognizable for a sideways observer, we reprogrammed the displays to be consistent with a vertical binocular separation and tested upright and sideways environments with sideways people. Our own subjective impression was that the ground plane appeared planar both when it was upright (relative to gravity) and when it was sideways (and therefore upright relative to the sideways observer). Under these circumstances, we expected to see larger angular expansion when the world and the observer were sideways (and thus apparent elevation in the sideways world was being estimated) than when only the observer was sideways, and the same direction appeared as azimuthal direction.

### 3.1. Methods

#### 3.1.1. Participants

Sixty-four observers were tested. All 64 were tested while lying on their side using a sideways forehead rest so that the scenes were viewed from a fixed position. Half of the participants made angular estimates in the upright world and then the sideways world; the other half saw them in the other order. Participants were required to have normal or corrected-to-normal vision in the absence of glasses.

#### 3.1.2. Apparatus and Design

The apparatus was similar to that in Experiments 1A and 1B, exempt that in order to provide a large field of view, polarizing filters were mounted so that they could extend vertically, with respect to the face, both up to the ridge about the eyes and down to the cheeks below. This was why participants with glasses could not be accommodated in this experiment.

The virtual environments and the experimental design were nearly the same as in Experiment 1B. The main change was that the participants completed the experiment while lying on their side on a couch with their head supported so as to lie horizontally. In addition, the angles used were reduced by 20% (i.e., 2.4° to 40.8°).

### 3.2. Results and Discussion

Angular gains were computed for each participant in each environment (mean *R*^2^ = 0.97), and used as the dependent measure in an ANOVA in which environment (upright or sideways) was a within-subject variable and order (upright first or sideways first) was a between-subject variable. As expected, the mean gain in the sideways-world condition, *M* = 1.44, 95% CI [1.31, 1.57], was reliably higher than the gain in the upright-world condition, *M* = 1.39, 95% CI [1.26, 1.51], *F*(1, 62) = 4.13, *p* = 0.046, *η^2^* = 0.003, though the effect was not large.

**Figure 3:**
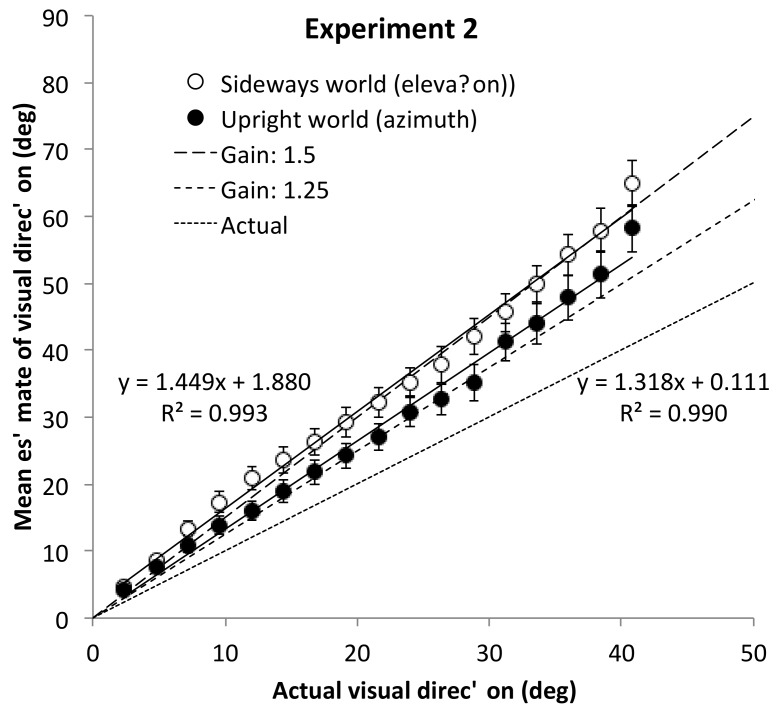
Summary data of the angular direction estimates in Experiment 2 by world orientation for those who did the relevant orientation first. For the sideways world, with sideways observers, where physical azimuth directions appeared as elevations above a ground plane, the angular estimates closely match the expected 1.5 gain normally found for perceived elevation direction.

Because we expected some contamination from one condition to the other, we also compared the effects in the two environments prior to contamination, and these are the data plotted in Figure 3. For the upright environment, when tested first, the mean angular gain (1.32, 95% CI [1.14, 1.51]) was not different from the pooled upright environment data (when measured first) from Experiments 1A and 1B (1.35, 95% CI [1.27, 1.44) with upright observers, *t*(62) = 0.33, *p* = 0.746, *d* = 0.08. This replicates prior observations suggesting that the visual ground plane, rather than the observer, defines the reference frame for differential angular expansion in azimuth and elevation [4,8]. Indeed, for the sideways environment, where we now expected a gain of 1.5, corresponding to the gain for elevation, the observed gain for those tested first in the sideways environment is consistent with this hypothesis, *M* = 1.52, 95% CI [1.33, 1.70]. The between-subjects comparison of these two groups was not statistically reliable, *t*(62) = 1.54, *p* = 0.128, *d* = 0.38, but the effect size observed was of medium strength. Contrasting the initial sideways condition of Experiment 2 with the pooled data from all the initial (gravitationally) upright conditions tested in Experiments 1 and 2, does indicate a reliable increase in this between-subject comparison, consistent with the one identified by the within-subject environment effect in the ANOVA above. Specifically, the mean in the sideways condition, *M* = 1.52, is marginally-reliably greater than the mean across the three upright conditions, *M* = 1.34, 95% CI [1.24, 1.44], *t*(94) = 1.90, *p* = 0.060, *d* = 0.41.

### 3.3. Conclusions

Presenting a sideways world to a sideways participant did cause a greater angular expansion in perception along the (gravitationally) horizontal axis, and specifically a gain that was almost exactly the predicted value of 1.5. That is, the mean gain, when the orientation of the ground plane defined the axis as an elevation axis, was essentially 1.5, as in prior reports of elevation estimates [6]. In contrast, the angular expansion observed along the horizontal axis for the upright virtual world, where the (gravitationally) horizontal axis corresponded to azimuth with respect to the ground plane, was reliably less than 1.5, but was consistent with the predicted value of 1.25. In these two conditions, the visual direction estimates were always along the same sagittal axis of the viewer, but the magnitude of the angular expansion corresponded to the axis defined by the virtual visual world, not by the retino-cortical direction nor by gravity.

## 4. General Discussion

**Figure 4:**
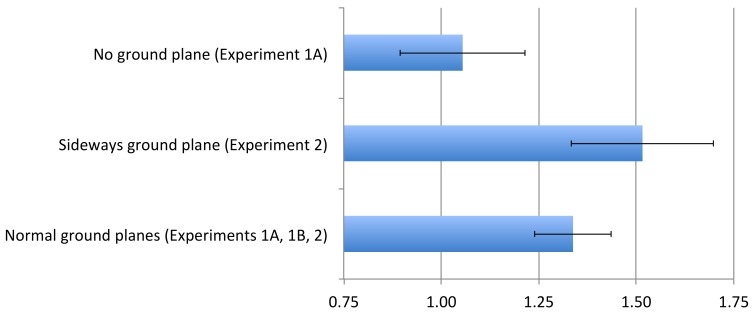
Mean angular gains for (gravitationally-defined) azimuth direction with 95% confidence intervals shown for three different visual worlds. Only data from the first world tested for each participant are shown, so comparisons are all between-subject and are uncontaminated by order effects.

We examined the role of the ground plane in differentiating the perception of angular direction in azimuth and elevation using virtual environments. A summary of the principal results is shown in Figure 4, based on the first world shown to a given participant, and based on a single-parameter model (angular gain). When the world was sideways and the ground plane was perceived as a ground plane (Experiment 2), judgments of physical azimuth (world elevation) were expanded with a gain of about 1.5, as has been reported in many other contexts for perceived angular elevation. Conversely, when the virtual environment was upright, angular gains were consistent in size, independent of the orientation of the observer consistent with the implications of prior reports [4]. As shown in Figure 4, the observed azimuthal gain for upright environments (~1.33) was not inconsistent with the value of 1.26 that has been measured previously [8] in an outdoor environment. Simply removing the ground plane, however, dropped the gain in judgments of azimuth nearly to 1.0.

Our most important two experimental results are that the presence or absence of a ground plane affected estimates of azimuth in Experiment 1A, and that the orientation of the ground plane affected estimates of physical azimuth in Experiment 2. Although these results cannot tell us why the ground plane has this effect, it supports the suggestion in previous research that a perceived visual ground surface plays a fundamental role in how the magnitude of deviation in visual direction relative to straight-ahead is perceived [4,8]. Why is angular expansion in perception linked to the presence and orientation of the ground? One possibility is that angular expansion is useful for the control of large-scale human actions. According to the formulation of Powers [16], action is the control of perception. Much as a watch-maker’s magnifying lens provides a useful distortion for behavioral control, perceiving a virtually magnified angular representation of the locomotor environment may also have utility for large-scale behavior on the ground [17,18]. The visual ground may serve as a contingent context for expanded encoding of angular variables.
